# Intramuscular blood flow and muscle oxygenation of the vastus lateralis response to intermittent incremental muscle contractions

**DOI:** 10.1113/EP091948

**Published:** 2024-12-10

**Authors:** Kazuma Izumi, Keisuke Yamamori, Keisho Katayama, Yutaka Kano, Noriko Tanaka, Hiroshi Akima

**Affiliations:** ^1^ Graduate School of Education and Human Development Nagoya University Nagoya Aichi Japan; ^2^ Research Center of Health, Physical Fitness and Sports Nagoya University Nagoya Aichi Japan; ^3^ Graduate School of Medicine Nagoya University Nagoya Aichi Japan; ^4^ Department of Engineering Science, Bioscience and Technology Program University of Electro‐Communications Chofu Tokyo Japan

**Keywords:** intramuscular blood flow, muscle oxygenation, power Doppler ultrasound

## Abstract

Power Doppler ultrasonography is used to measure blood flow within a given muscle, otherwise known as intramuscular blood flow. However, it is not fully understood how intramuscular blood flow and muscle oxygenation change with repetitive muscle contraction. The present study was conducted to assess changes in intramuscular blood flow and muscle oxygenation of the vastus lateralis (VL) during intermittent and incremental contractions. Fifteen healthy male subjects (21.7 ± 2.6 years) performed intermittent (5 s contraction, 5 s relaxation) and incremental isometric knee extensions at 30%, 40%, 50%, 60% and 70% of maximal voluntary contraction (MVC) until task failure. Intramuscular blood flow and muscle oxygen saturation ($S_{tO_{2}}$) were simultaneously measured using power Doppler ultrasonography and near‐infrared spectroscopy, respectively, from the right VL of the mid‐thigh. Intramuscular blood flow was increased from 0.5 ± 0.5% at rest to 13.9 ± 9.5% at task failure. Intramuscular blood flow significantly increased from rest to 30% and 40% MVC (*P* = 0.001), and $S_{tO_{2}}$ significantly decreased from 30% to 70% MVC (*P* = 0.004). These results indicate that intramuscular blood flow and $S_{tO_{2}}$ show different patterns of change, suggesting that the contribution of intramuscular blood flow to oxygen supply decreases within the VL at moderate and higher exercise intensities.

## Abstract (highlights)

- **What is the central question of this study?**
  How do intramuscular blood flow and muscle oxygenation change with intermittent and incremental muscle contractions?
- **What is the main finding and its importance?**
  Intramuscular blood flow reached a plateau above moderate exercise intensities, but muscle oxygen saturation continued to decrease. These results indicate that the contribution of intramuscular blood flow to oxygen supply varies with exercise intensity.

## 1 INTRODUCTION

An increase in blood flow is crucial for exercise performance as it supplies oxygen and eliminates metabolic by‐products. Total limb blood flow increases in proportion to exercise intensity during dynamic (Andersen & Saltin, 1985; Rådegran, 1997) and intermittent isometric knee extensions (Osada et al., 2015). When oxygen supply and demand are matched, exercise can be sustained by enhanced ATP production in the mitochondria. Conversely, when oxygen supply and demand are not matched, the limited non‐oxidative energy reserves are rapidly consumed, accelerating fatigue (Poole et al., 1988).

Both invasive and non‐invasive methods, such as plethysmography Hunter et al., 2008, pulse‐wave Doppler (Osada et al., 2015; Rådegran, 1997), magnetic resonance imaging (Wigmore et al., 2004), positron emission tomography (PET) (Kalliokoski et al., 2000), and the combination of near‐infrared spectroscopy (NIRS) and indocyanine green (Habazettl et al., 2010) have been used to quantify blood flow in the extremities during exercise. Pulse‐wave Doppler could be an ideal technique to measure blood flow through the femoral artery during knee extension exercise in humans (Osada et al., 2015; Rådegran, 1997). However, given that knee extension exercise increases blood flow not only in the quadriceps muscle but also in the skin, adipose tissue, and bone of the thigh (Heinonen, Kemppainen et al., 2014), the increase in femoral artery blood flow may not fully reflect the increase in blood flow in the quadriceps muscle. The blood flow distribution to these tissues changes according to sympathetic vasoconstriction (Heinonen et al., 2013). As such, it is difficult to assess the blood flow within specific active muscles using pulse‐wave Doppler. The changes in blood flow within each muscle are unclear because PET studies have shown the distribution of blood flow is heterogeneous among quadriceps muscles (Kalliokoski et al., 2000). PET might be a useful alternative technique to quantify blood flow to a specific muscle region (Kalliokoski et al., 2000); however, radiation exposure should be considered. Consequently, the change and distribution of blood flow within a given muscle, otherwise termed intramuscular blood flow, during exercise are poorly understood due to the lack of available methods to measure it non‐invasively in humans.

Power Doppler ultrasonography is a non‐invasive technique for quantitatively assessing the blood flow within a specific region, unlike conventional methodologies. Power Doppler ultrasonography can be used to measure the frequency shift derived from the movement of erythrocytes and encodes an estimate of the integrated Doppler power spectrum (Rubin et al., 1994). The intensity of the Doppler signal, which is correlated with the number of erythrocytes causing the Doppler shift, is represented by the brightness of the colour signal. As the intensity of the Doppler signal increases, the brightness of the colour displayed within the vessels becomes lighter (Rubin et al., 1994). As far as we know, only a few studies have examined intramuscular blood flow during intermittent muscle contraction (Dietz et al., 2020; Dori et al., 2016; Heres et al., 2018); however, the exercise intensity was not properly set in those studies. Additionally, previous studies did not analyse the brightness of power Doppler signals, which represents blood volume (Dietz et al., 2020; Dori et al., 2016; Heres et al., 2018). Therefore, it is essential to strictly set exercise intensity when imposing an exercise load on participants and to analyse data considering the brightness of the power Doppler signals.

NIRS can be used to measure muscle oxygen saturation ($S_{tO_{2}}$), which reflects the balance between oxygen supply and extraction (McCully & Hamaoka, 2000). Both blood flow and oxygen extraction increase with increasing exercise intensities, even during submaximal exercise, in skeletal muscles (Heinonen, Kudomi et al., 2014). This implies that the relationship between exercise intensity and oxygen dynamics is more complex than a simple linear decrease in $S_{tO_{2}}$. While $S_{tO_{2}}$ linearly decreases with increasing workload (Grassi et al., 1999), it is essential to consider the concurrent increases in blood flow and oxygen extraction that occur, ensuring adequate oxygen delivery during submaximal exercise intensities. When combined with power Doppler ultrasonography, it can show the dynamics of oxygen supply and demand within the working muscle, simultaneously. This approach could provide valuable insights into improvements in the peripheral circulation and muscle dysfunction, particularly in elderly individuals and those with diabetes mellitus. To the best of our knowledge, no studies have measured intramuscular blood flow and muscle oxygenation simultaneously during incremental repetitive contractions.

The purpose of this study was to assess the changes in intramuscular blood flow and muscle oxygenation during intermittent and incremental muscle contractions. We hypothesized that intramuscular blood flow would increase 2015 and $S_{tO_{2}}$ would decrease linearly with an increase in exercise intensity (Grassi et al., 1999).

## 2 METHODS

### 2.1 Ethical approval

This study was approved by the Ethics Committee of the Research Center of Health, Physical Fitness & Sports at Nagoya University (21‐05) and was performed in accordance with the *Declaration of Helsinki*. Before the experiments, the purpose, risks and benefits of this study were explained, and written informed consent was obtained from all participants.

### 2.2 Experimental procedure

Fifteen healthy male subjects participated in this study (age, 21.7 ± 2.6 years; height, 172.6 ± 5.5 cm; weight, 64.1 ± 9.9 kg). All participants visited the laboratory on two separate days with an interval of 1 week. On the first visit, the participants practiced maximal voluntary contraction (MVC) and familiarized themselves with the intermittent and incremental contraction tasks. On the second visit, the participants performed MVC measurement and intermittent and incremental contractions until task failure following warm‐up with submaximal contractions.

### 2.3 MVC measurement

**FIGURE 1:**
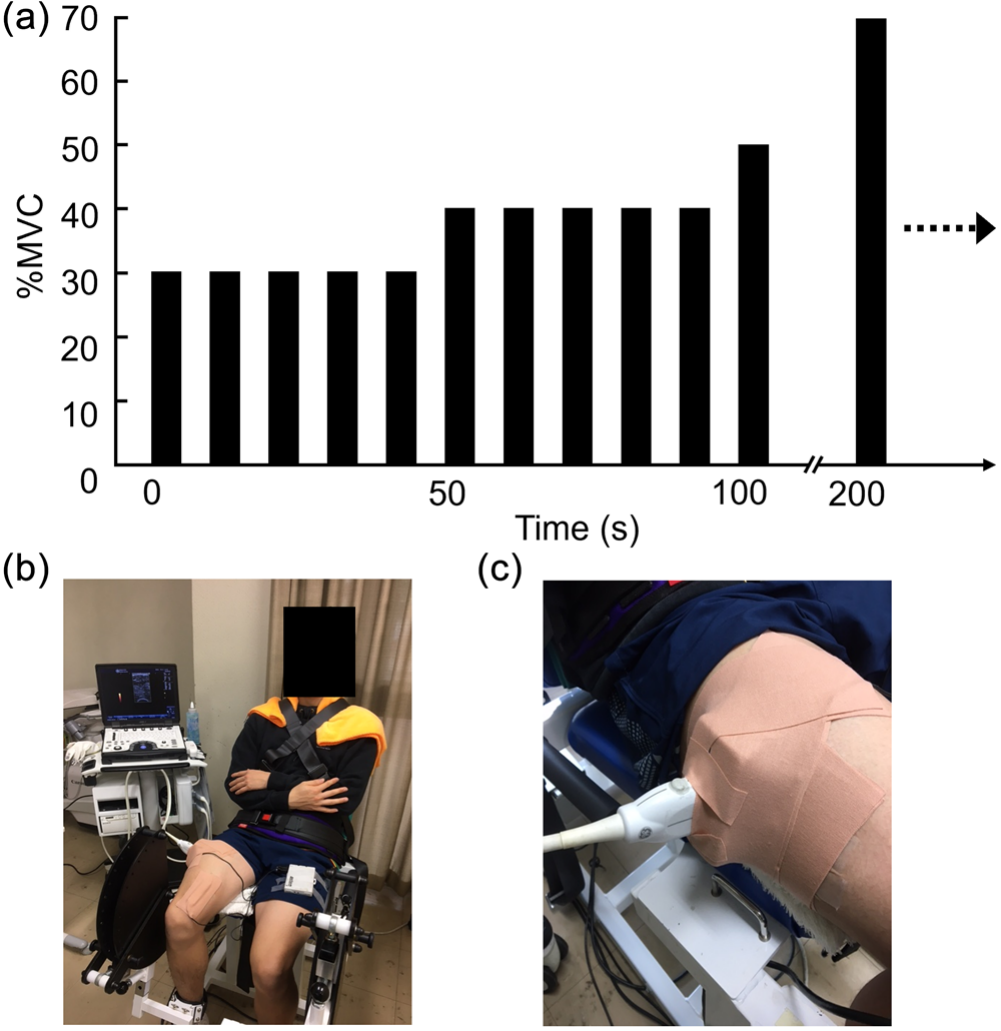
Protocols for intermittent and incremental exercise (a), experimental setting (b), and ultrasound probe fixation (c). MVC, maximal voluntary contraction.

The participants performed an isometric knee extension MVC with their right leg using a custom‐designed dynamometer (M‐12297‐3; Takei Scientific Instruments Co., Ltd, Niigata, Japan) mounted on a force transducer (LTZ‐100KA; Kyowa Electronic Instruments Co., Ltd, Tokyo, Japan), as reported previously (Watanabe & Akima, 2009). During the knee extension tasks, the hip, chest and ankle were tightly fixed to the seat using straps. The hip joint and knee joint were flexed at angles of 70° and 90°, respectively (Figure 1). The participants were asked to hold their arms crossed in front of their chest. The MVC test was performed three times with ≥2 min rest after submaximal contractions. The MVC test consisted of three phases: the rising phase (2−3 s), the sustained phase (≥3 s) and the relaxation phase (Watanabe & Akima, 2009). The supervisors encouraged the participants to exert vigorous maximal effort. The knee extension force was recorded at 400 Hz using an analog‐to‐digital converter (PowerLab 16SP; ADInstruments, Melbourne, Victoria, Australia) and stored in a personal computer (Mac Mini; Apple Inc., Cupertino, CA, USA). For each contraction, the MVC force was determined as the peak value. An extra trial was conducted if there was a difference of >5% between the two greatest exerted forces between trials.

### 2.4 Intermittent and incremental knee extensions protocol

After rest for 10 min following the MVC measurement, the participants performed intermittent (5 s contraction, 5 s relaxation) and incremental isometric knee extensions. The exercise began at 30% of MVC and increased by 10% every five contractions to 70% of MVC until task failure (Figure 1). Task failure was defined as the point at which the participants had completed 30 muscle contractions at 70% of MVC or when the exerted force had fallen below the target force for three consecutive exertions. During the exercise, the participants were provided with real‐time force feedback on a computer monitor placed in front of the participant with the target force shown and were strongly encouraged by the supervisors. Ten seconds after cessation of the fatiguing task, a second MVC measurement was made.

### 2.5 Intramuscular blood flow measurement

Power Doppler ultrasonography (LOGIQ e Premium; GE Healthcare, Wauwatosa, WI, USA) with a 12 MHz linear‐array probe (probe width, 3.8 cm) was used to assess intramuscular blood flow in the vastus lateralis (VL) during intermittent and incremental muscle contractions. The probe was placed perpendicular to the estimated longitudinal axis of the VL on the skin using a custom‐made styrene frame (Figure 1). Transverse images were obtained at the mid‐thigh. Sufficient transducer gel was applied to the probe to provide acoustic contact without depression of the dermal surface. The power Doppler ultrasonography settings were held constant over all measurements with the following acquisition parameters: frequency, 6.3 MHz; gain, 30.5 dB; depth, 7 cm. The Doppler window was configured to display the entire image field. Power Doppler images displayed on the ultrasonography monitor screen were stored on a personal computer (LAVIE; NEC Corp., Tokyo, Japan) via a capture device (DVI2USB 3.0; Epiphan Video, Ottawa, ON, Canada) in AVI format at a sampling rate of 20 frames/s.

**FIGURE 2:**
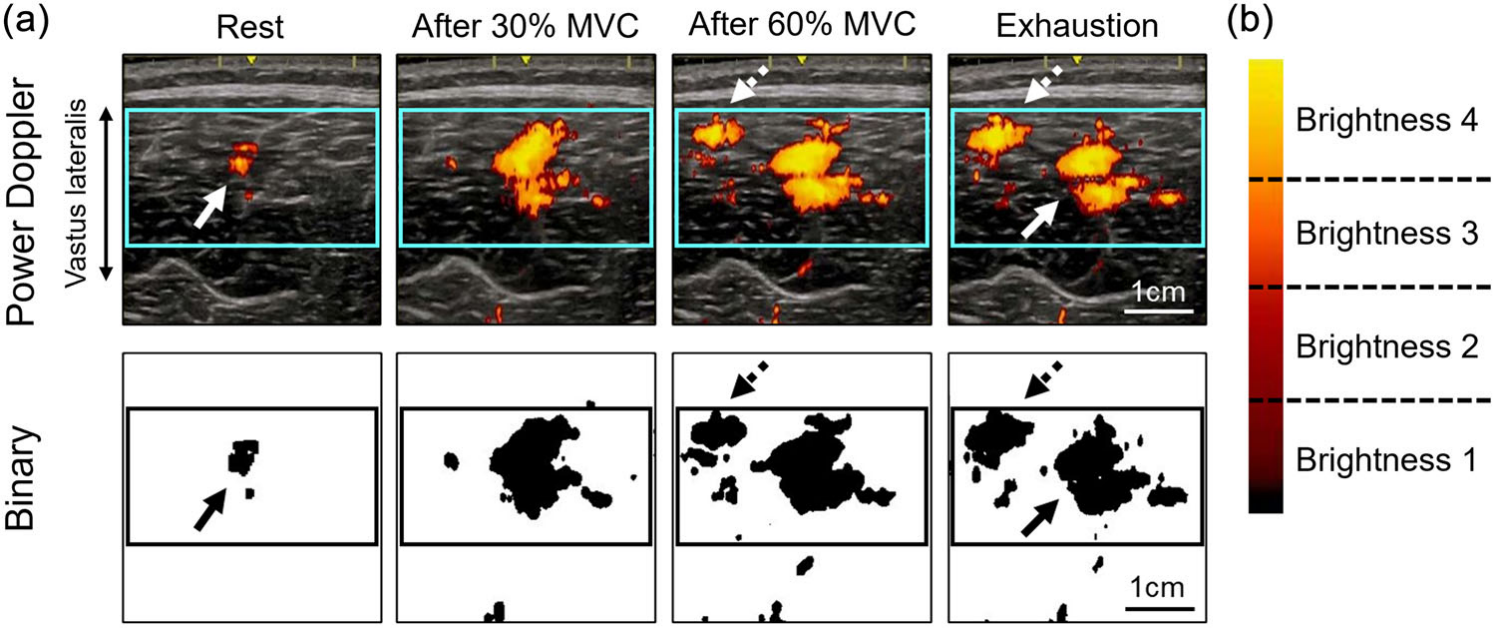
Representative power Doppler images of the vastus lateralis at the mid‐thigh (upper row in (a)) and binary images used to calculate intramuscular blood flow (lower row in (a)) and brightness division (b). Intramuscular blood flow is represented by the relative area of binarized power Doppler signals in the region of interest. A continuous arrow indicates the vessels detected at rest, and the dashed arrows indicate the vessels detected due to increased blood flow. The outlined part is the region of interest. The power Doppler signal was divided into four parts according to the brightness, and the relative area of each brightness level was calculated.

Intramuscular blood flow was analysed using custom‐designed power Doppler signal‐measuring software (S‐22028 version 1.0.3; Takei Scientific Instruments Co., Ltd). This software was developed to calculate the pixel count of the power Doppler signal within the image. Intramuscular blood flow was assessed by the relative area of the power Doppler signal in the region of interest using the following equation (Figure 2a) (Dori et al., 2016): intramuscular blood flow (%) = (cross‐sectional area of power Doppler signal) / (cross‐sectional area of region of interest) × 100.

The region of interest in the VL was determined using rectangular selections at the frame in which muscle contraction was not exerted, and there was no movement artifact during muscle contraction. The selected area included as much muscle as possible but avoided visible fascia. Additionally, the power Doppler signal was divided into quartiles based on its brightness (Figure 2b), and the relative area of the signal at each brightness level (brightness levels, 1−4) was calculated. The brightness of the signal varies with blood flow; the higher the blood flow, the lighter the signal (brightness level 4 indicates the area with the highest blood flow) (Rubin et al., 1994).

### 2.6 Muscle oxygenation measurement

Muscle oxygenation of the VL was recorded using the NIRS device (Hb14; Astem Co., Ltd, Kanagawa, Japan) with a dual‐wavelength (770 and 830 nm) light‐emitting diode, as previously described (Akima & Ando, 2017). The probe of the NIRS system consisted of one light source and two photodiode detectors, and the optode distances were 2 and 3 cm. Oxyhaemoglobin/myoglobin (oxy‐Hb/Mb), deoxyhaemoglobin/myoglobin (deoxy‐Hb/Mb) and total haemoglobin/myoglobin (total Hb/Mb) were determined by measuring the light attenuation at wavelengths of 770 and 830 nm and were analysed using algorithms based on a modified Beer–Lambert law (Kime et al., 2010). The thickness of subcutaneous fat (that is, the path length) in the region at which the NIRS signal was determined using B‐mode ultrasonography (LOGIQ e Premium; GE Healthcare). This measurement was required as the input to run the NIRS program on a personal computer (ENVY; Hewlett‐Packard Japan, Tokyo, Japan), and was used by the software to determine the relative change in Hb/Mb and the absolute $S_{tO_{2}}$ values. The absolute $S_{tO_{2}}$ values were provided by the NIRS system and were calculated using the relative absorption coefficients obtained from the light attenuation slope over a distance measured at two focal points from the light emission. The NIRS probe was attached perpendicular to the estimated longitudinal axis of the VL at the distal neighbour to the ultrasound probe used to measure intramuscular blood flow. The probe was fixed using double‐sided adhesive tape and covered with elastic therapeutic tape to prevent interference from undesired light. The NIRS signals were sampled at 2 Hz and transferred to a personal computer (ENVY; Hewlett‐Packard Japan) via a wireless connection (Bluetooth 2.0).

### 2.7 Electrocardiogram measurement

Electrocardiogram was recorded by three‐lead electrocardiography (AB‐621; Nihon Kohden, Tokyo, Japan) and stored in a personal computer (Mac Mini; Apple Inc.) at 400 Hz via an analog‐to‐digital converter. Heart rate (HR) was determined from the R‐R interval.

### 2.8 Data analysis

The resting values of all parameters were averaged over 1 min just before the exercise protocol. The values for intramuscular blood flow and $S_{tO_{2}}$ were averaged during the relaxation phases at each exercise intensity, and the HR value was averaged over 3 s periods. However, we excluded 1 s before and after during the 5 s relaxation phase of the exercise protocol to avoid contraction‐induced artifacts. The exhaustion values were averaged over the last three contractions in which the participants were unable to maintain the target force. The ratio of the absolute change from baseline (Δ) in intramuscular blood flow to deoxy‐Hb/Mb (Δintramuscular blood flow/Δdeoxy‐Hb/Mb) was calculated to assess the relationship between oxygen supply (intramuscular blood flow) and muscle deoxygenation (deoxy‐Hb/Mb). A segmented regression model in R‐studio (http://www.rstudio.com) was used to determine the inflection point of Δintramuscular blood flow/Δdeoxy‐Hb/Mb.

### 2.9 Statistical analysis

All values are reported as the mean ± SD. Student's paired *t*‐test was used to compare pre‐MVC with post‐MVC. One‐way ANOVA with repeated measures was used to compare intramuscular blood flow, $S_{tO_{2}}$ and HR among the different exercise intensities. When ANOVA showed a significant difference, Bonferroni's *post hoc* test was performed. Two‐way (time × brightness) ANOVA with repeated measures was used to assess the difference between each brightness. When interactions or main effects were found, Tukey's *post hoc* test was used to identify significant differences. Statistical analyses were performed using IBM SPSS Statistics software (version 27, IBM Corp., Tokyo, Japan). The level of significance was set at *P* < 0.05.

## 3 RESULTS

Before the repetitive knee extension exercise, MVC was 186.2 ± 49.0 N m. After the exercise, MVC decreased to 147.1 ± 38.9 N m, showing a significant 20% reduction after exhaustion (*P* < 0.0001). The time to exhaustion was 349 ± 94 s. The mean values for achieved %MVC and HR during intermittent and incremental exercise are shown in Table 1. HR significantly increased from 80.1 ± 11.1 to 134.1 ± 19.2 bpm with exercise intensity (*P* = 0.018).

**TABLE 1:** Mean values for achieved %MVC and HR during intermittent and incremental exercise.

		%MVC					
Variable	Rest	30%	40%	50%	60%	70%	Exhaustion
Achieved %MVC	0.0 ± 0.0	29.5 ± 0.8 *	39.3 ± 0.7 *	49.4 ± 0.7 *	59.3 ± 0.7 *	69.0 ± 1.7 *	65.6 ± 3.7 *
Heart rate (bpm)	80.1 ± 11.1	89.6 ± 11.3 †	92.0 ± 11.0	99.8 ± 10.8 †	110.0 ± 13.1 †	120.0 ± 15.5 †	134.1 ± 19.2 *

Values are means and SD; *n* = 15. *P < 0.05, †*P* < 0.01 vs. one‐lower intensity. bpm, beats per minute; HR, heart rate; MVC, maximal voluntary contraction.

Intramuscular blood flow significantly increased approximately 30‐fold from 0.5 ± 0.5% at rest to 13.9 ± 9.5% at exhaustion. Figure 2a shows representative transverse power Doppler images (upper panel) and the binary images (lower panel) used to assess the intramuscular blood flow of the VL at rest, after 30% MVC, after 60% MVC and at exhaustion.

**FIGURE 3:**
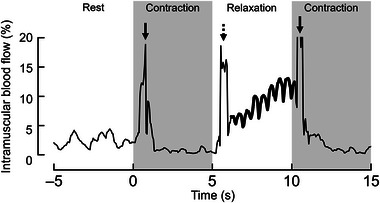
Representative time course of intramuscular blood flow at the start of the exercise protocol. Intramuscular blood flow was represented by the relative area of power Doppler signals in the region of interest and was averaged over the value drawn with a bold line. The arrows point to the rapid increase in value caused by the motion artifacts at muscle contraction (continuous) and relaxation (dashed).

Figure 3 shows a representative time course of change in intramuscular blood flow at the start of the exercise protocol. At the onset of contraction, muscle contraction‐induced artifacts (continuous arrows) were recorded, and then intramuscular blood flow turned markedly lower during the contraction phase. At the onset of the relaxation phase, muscle relaxation‐induced artifacts (dashed arrow) were observed. Then, an increase in blood flow was observed during about 4 s and between 6 and 10 s (Figure 3).

**FIGURE 4:**
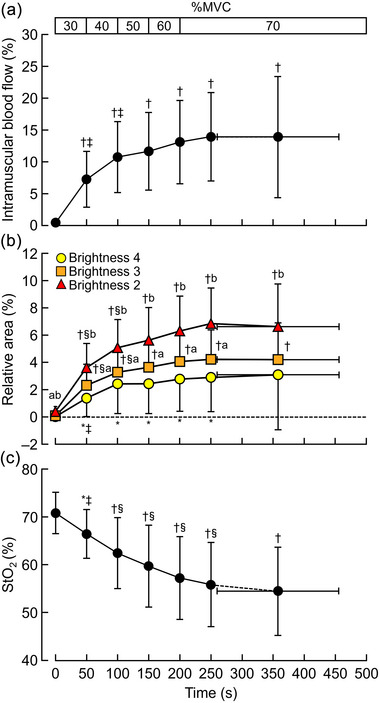
The changes in intramuscular blood flow (a), brightness (b), and $S_{tO_{2}}$ (c) during intermittent and incremental contractions. Intramuscular blood flow was represented by the relative area of power Doppler signals in the region of interest. The data in (b) show the value of intramuscular blood flow (a) divided into four brightness levels as shown in Figure 2b. Brightness level 1 is not shown here because it was not detected. *n* = 15. MVC, maximal voluntary contraction; $S_{tO_{2}}$, muscle oxygen saturation. *P < 0.05, †*P* < 0.01 vs. rest. ‡*P* < 0.05, §*P *< 0.01 vs. one‐lower intensity. ^a^ *P* < 0.01 vs. brightness level 4 at the same intensity. ^b^ *P* < 0.01 versus brightness level 3 at the same intensity.

Figure 4 shows the changes in intramuscular blood flow (Figure 4a), brightness (Figure 4b) and $S_{tO_{2}}$ (Figure 4c) during intermittent and incremental muscle contractions. Compared with the resting condition, intramuscular blood flow significantly increased at all exercise intensities (*P* = 0.002). Intramuscular blood flow significantly increased from 30% to 40% MVC (*P* < 0.0001) when comparing the changes between consecutive intensities. The power Doppler signal was divided into four equal parts by brightness (1−4) (Figure 2b), and the change in the relative area within the region of interest of the signal for each brightness was analysed. No signal corresponding to brightness 1 was detected. There were significant brightness‐by‐time (exercise intensity) interactions (*F*_12,168 _= 12.985, *P* < 0.0001). Significant brightness (*F*_2,28_ = 55.421, *P* < 0.0001) and time effects (*F*_6,84_ = 21.136, *P* < 0.0001) were observed. Brightness levels 2, 3 and 4 significantly increased at all exercise intensities (*P* = 0.042); the only exception was that brightness level 4 did not increase at exhaustion compared to rest (*P* = 0.264). Brightness levels 3 and 4 increased from 30% to 40% MVC compared to the value at one‐lower intensity (*P* = 0.001). The comparisons between brightness levels at each exercise intensity showed that brightness level 3 was significantly higher than brightness level 4 (*P* = 0.009) at all exercise intensities, except exhaustion (*P* = 0.093), and brightness level 3 was significantly higher than brightness level 2 (*P* = 0.006) at all exercise intensities. $S_{tO_{2}}$ significantly decreased at all exercise intensities compared to rest (*P* = 0.027), and it significantly decreased from 30% to 70% MVC (*P* = 0.004).

**FIGURE 5:**
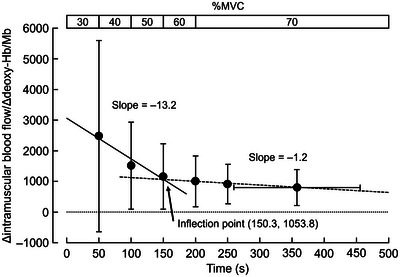
The change in the ratio of absolute changes (Δ) from baseline in intramuscular blood flow to deoxy‐Hb/Mb (Δintramuscular blood flow/Δdeoxy‐Hb/Mb). An inflection point was identified by calculating the intersection of the segmented regression lines. The regression lines below (continuous line) and above (dashed line) the inflection point are indicated by the equations *y* = −13.22*x* + 3040.02 (*r* ^2^ = 0.93) and *y* = −1.23*x* + 1238.95 (*r* ^2^ = 0.98), respectively. *n* = 15. Deoxy‐Hb/Mb, deoxyhaemoglobin/myoglobin; MVC, maximal voluntary contraction.

Figure 5 shows the Δintramuscular blood flow/Δdeoxy‐Hb/Mb. The inflection point occurred at 117.7 s after exercise began (i.e., during 50% MVC). The regression line is represented according to the following equations:

$$\begin{array}{c} \text{Left side regression line}:y=-13.22x+3040.02(r^{2}=0.93).\\ \text{Right side regression line}:y=-1.23x\;+\;1238.95(r^{2}\;=\;0.98). \end{array}$$

## 4 DISCUSSION

The main finding of this study was that intramuscular blood flow reached a plateau above moderate‐intensity exercise. In contrast, muscle oxygenation continued to decrease with the elevation of exercise intensity, which means that oxygen demand exceeded supply during fatiguing exercise. We observed a significant increase in intramuscular blood flow and a significant decrease in $S_{tO_{2}}$ from the beginning of exercise until task failure. Moreover, intramuscular blood flow significantly increased from rest to 40% MVC, and then plateaued until task failure. However, $S_{tO_{2}}$ significantly decreased from rest to 70% MVC, which was a slightly different pattern of intramuscular blood flow.

It is difficult to quantify the amount of blood flow within a specific region of muscle during exercise non‐invasively and with high temporal resolution. To overcome these problems, we used power Doppler ultrasonography to assess blood flow within the VL during repetitive and incremental force exertion tasks. We found that intramuscular blood flow represented by the relative area of power Doppler signal within the region of interest was almost zero, with a value of 0.5 ± 0.5% before exercise. This may be because resting intramuscular blood flow was extremely low compared to during exercise, which could have been a main reason for the intramuscular blood flow not being detected. Then, it significantly and non‐linearly increased to 13.9 ± 9.5% at task failure, which is roughly 30‐fold the resting value. This result is consistent with previous studies using the same technique. For instance, intramuscular blood flow in the forearm flexors increased from 1.2% to 7.3% following intermittent maximal handgrip exercise (Dori et al., 2016), and blood flow within the VL increased to 17% from rest during cycling exercise (Heres et al., 2018). Comparing the results of this study with those of previous studies (Dori et al., 2016; Heres et al., 2018) is challenging due to differences in the experimental conditions, such as the type of equipment, measurement sites and exercise protocols. This increase in intramuscular blood flow could be attributed to both an increase in blood flow in the vessels already detected at rest and newly detected blood flow that was not previously found at rest (Dori et al., 2016).

We identified a successful method to make it easier to see the differences in the magnitude of power Doppler signals as colour‐coded levels (Figure 2), which has not been reported previously. The signal was divided into quartiles (levels 1–4, with the higher level indicating more blood flow) based on the brightness, and we assessed changes in the relative area of the signal at each brightness level in the region of interest. Blood flow affects the brightness of the signal; the greater the blood flow, the lighter the signal (Rubin et al., 1994), with brightness level 4 representing the region with the highest blood flow. Consequently, we observed significant interactions between brightness and time (exercise intensity). At almost all exercise intensities, brightness level 3 was significantly greater than brightness level 4, and brightness level 2 was significantly greater than brightness level 3 (Figure 4b). Thus, the greatest increase in the low‐intensity signal may reflect an increase in the near outer portion of the signal, that is, increased intramuscular blood flow due to vasodilatation.

We measured intramuscular blood flow during intermittent and incremental isometric knee extensions due to motion artifacts in dynamic exercise with power Doppler ultrasonography. Increases in intramuscular blood flow reached plateau at above 50% MVC during intermittent and incremental knee extension (Figure 4a). Previous studies have showed a positive correlation between leg blood flow and exercise intensity during dynamic exercise (Andersen & Saltin, 1985; Rådegran, 1997), which contradicts the present study. Quadriceps blood flow is significantly higher during dynamic than isometric exercise (Laaksonen et al., 2003), suggesting the type of exercise influences the results. However, exercise type likely did not affect results, since a linear relationship between blood flow and the level of contraction was found even with intermittent isometric exercise (Osada et al., 2015; Wigmore et al., 2006). Potentially, the discrepancy of the results between this study and previous studies (Osada et al., 2015; Wigmore et al., 2006) has been explained by measurement techniques and/or the characteristics of the participants. Unlike these previous studies (Osada et al., 2015; Wigmore et al., 2006), previous research utilizing a combination of NIRS and indocyanine green, has demonstrated that blood flow in individual muscles reaches plateau at submaximal intensity during incremental exercise (Boushel et al., 2002; Habazettl et al., 2010). Blood flow heterogeneity within the quadricep muscles decreases with increasing workload during intermittent isometric knee extension exercise (Heinonen et al., 2007). These findings suggest that during high‐intensity exercise, motor units with lower metabolic efficiency could be engaged to meet the increased force demands, and additional enhancements in limb blood flow are directed toward these newly activated muscle areas (Boushel et al., 2002; Habazettl et al., 2010). The increase in intramuscular blood flow reaching a steady state at intensities above 50–70% MVC in the present study may reflect blood flow heterogeneity. Thus, while blood flow linearly increases with exercise intensity when evaluating active muscles at the level of the whole limb (Osada et al., 2015; Wigmore et al., 2006), our study proposes that, unlike changes in conduit arterial blood flow, the relationship between exercise intensity and blood flow may not necessarily be linear when assessing individual active muscles.

The strength of this study is that we semi‐quantified intramuscular blood flow depending on the exercise intensity (Table 1). Contrary to the hypothesis, intramuscular blood flow plateaued above 50% MVC, and $S_{tO_{2}}$ decreased even at the intensities at which intramuscular blood flow (oxygen supply) reached a submaximal plateau. These results indicate that the contribution of intramuscular blood flow to oxygen delivery decreased at above moderate intensities, as $S_{tO_{2}}$ decreased despite the plateau in intramuscular blood flow. It is important to note that both blood flow and oxygen extraction increase with exercise intensity, even during submaximal efforts, as observed in skeletal muscle. This suggests a more complicated interaction between blood flow and oxygenation during exercise, where adequate blood flow is maintained across varying intensities to meet the metabolic demands of the working muscle. However, blood flow and oxygen extraction increase with increasing exercise intensity in skeletal muscle, even during submaximal exercise, without indicating inadequate blood flow (Heinonen, Kudomi et al., 2014). One possibility is that, at intensities above 50% MVC, the accumulation of metabolic by‐products and the decrease in intramuscular pH led to more oxygen extraction due to the Bohr effect. Oxidative metabolism of the gastrocnemius muscle accelerated above 40% MVC of intermittent and incremental exercise (Homma et al., 2005). This observation may explain the enhancement of oxygen extraction within the working muscle at moderate or higher intensities.

NIRS signals may be affected by adipose tissue (Heinonen, Kemppainen et al., 2014; Heinonen et al., 2016) and skin blood flow (Tew et al., 2010). Adipose tissue blood flow increases in response to low‐intensity exercise, but levels off when exercise intensity is further increased (Heinonen, Kemppainen et al., 2014; Heinonen et al., 2016). The change in adipose tissue blood flow is probably due to the vasoconstriction of adipose tissue arterioles caused by increased sympathetic nervous activity. This process redistributes total limb blood flow to working muscles that depend on oxygen delivery during exercise (Heinonen, Kemppainen et al., 2014; Heinonen et al., 2016). Therefore, while $S_{tO_{2}}$ signal is reduced, it may not be fully reflected in intramuscular blood flow.

To understand the coupling between time course changes in intramuscular blood flow and muscle deoxygenation, we assessed the ratio of oxygen supply to extraction (Δintramuscular blood flow/Δdeoxy‐Hb/Mb) (Figure 5). The ratio decreased with an increase in exercise intensity, and an inflection point was identified at 117 s after the start of exercise (during 50% MVC). This indicates that the balance between oxygen supply and demand shifted to moderate intensity. Below 40% MVC, oxygen supply, because of the increase in intramuscular blood flow, exceeded the oxygen demand via an increase in deoxy‐Hb/Mb. However, above 50% MVC, the oxygen supply and demand matched (Figure 5). A previous study by Ferreira et al. (2007) showed faster muscle blood flow kinetics than muscle oxygen consumption at a lower work rate, which supports the results of the present study. The rapid increase in intramuscular blood flow could be due to the muscle pump action and/or rapid vasodilatation (Shoemaker & Hughson, 1999; Tschakovsky & Sheriff, 2004).

This study has some limitations. First, intramuscular blood flow was measured at only mid‐thigh of the VL. Therefore, it may not be representative of changes across the whole muscle. We need to measure blood flow at different sites due to heterogeneous neuromuscular activation patterns (Watanabe & Akima, 2010) and blood flow (Heinonen et al., 2015; Kalliokoski et al., 2006) among the quadriceps muscles. Future research should examine blood flow dynamics in active muscles other than the VL. Second, intramuscular blood flow measurements are expressed as a relative value (the relative area of power Doppler signal within the region of interest), and we compared exercise response with baseline. Another drawback is that we recruited only young healthy males. The changes in muscle perfusion and fatigability tolerance might differ between men and women during and after exercise (Hunter, 2014). Future research is needed to demonstrate the effect of physical characteristics on intramuscular blood flow.

In conclusion, we assessed the changes in intramuscular blood flow and muscle oxygenation during intermittent and incremental muscle contractions. The increase in intramuscular blood flow reached a steady state at 40% MVC, while $S_{tO_{2}}$ continued to decrease. The ratio of the increase in intramuscular blood flow to the increase in deoxygenation decreased with an increase in exercise intensity, with an inflection point at 50% MVC. These results indicate that intramuscular blood flow and $S_{tO_{2}}$ show different patterns of change, suggesting that the contribution of intramuscular blood flow to oxygen supply decreases within the VL at moderate and higher exercise intensities.

## AUTHOR CONTRIBUTIONS

Conception of design of the work: Kazuma Izumi, Keisuke Yamamori, Keisho Katayama, Noriko Tanaka and Hiroshi Akima Acquisition, analysis, or interpretation of the data for the work: Kazuma Izumi, Keisuke Yamamori, Keisho Katayama, Yutaka Kano, Noriko Tanaka and Hiroshi Akima Drafting of the work or revising it critically for important intellectual content: Kazuma Izumi, Keisuke Yamamori, Keisho Katayama, Yutaka Kano, Noriko Tanaka and Hiroshi Akima All authors approved the final version of the manuscript and agree to be accountable for all aspects of the work in ensuring that questions related to the accuracy or integrity of any part of the work are appropriately investigated and resolved. All persons designated as authors qualify for authorship, and all those who qualify for authorship are listed.

## CONFLICT OF INTEREST

None declared.

## Data Availability

The datasets generated and analysed during the current study are available from the corresponding authors upon reasonable request.
